# A comparison of quality of sleep between patients with chronic kidney disease not on hemodialysis and end-stage renal disease on hemodialysis in a developing country

**DOI:** 10.1080/0886022X.2017.1361836

**Published:** 2017-08-14

**Authors:** Salman T. Shafi, Tahir Shafi

**Affiliations:** Department of Nephrology, Sharif Medical and Dental College, Lahore, Pakistan

**Keywords:** Quality of sleep, chronic kidney disease, hemodialysis, comparison, developing country

## Abstract

Few studies have compared quality of sleep between pre-dialysis chronic kidney disease (pre-dialysis CKD) patients and end-stage renal disease patients on dialysis (ESRD) and have found inconsistent results. Objective of this study is to compare quality of sleep between patients with pre-dialysis CKD and ESRD in a developing country. This study was conducted in an out-patient department and hemodialysis unit of a tertiary care facility. Patients included had either pre-dialysis CKD or ESRD. Assessment of quality of sleep was done using Pittsburgh sleep quality index (PSQI). A total of 152 patients were included in the study. Out of these patients, 79 (52%) had ESRD and 73 (48%) had pre-dialysis CKD. Median PSQI score was 6 (IQR 3–8.8). Poor sleep quality (PSQI ≥5) was present in 100 (65.8%) patients. Only hemoglobin (*β* = −0.39, *p* < .01), depression (*β* = 0.56, *p* < .01) and history of cardiovascular disease (*β* = 0.22, *p* < .01) were associated with PSQI global score in a multiple linear regression analysis. There was no significant association between ESRD vs. pre-dialysis CKD and PSQI global scores and no significant co-relation between eGFR and global PSQI score (*r* = −0.34, *p* value .80) in pre-dialysis CKD patients. Poor sleep quality is common in patients with CKD including hemodialysis patients in a developing country, which is independent of kidney function in non-dialysis patients. There is no difference in quality of sleep between pre-dialysis CKD and ESRD patients.

## Introduction

Chronic kidney disease (CKD) especially end-stage renal disease (ESRD) is associated with reduced sleep quality, sleep disordered breathing, excessive day time sleepiness and periodic leg movement [1–5].

Several studies have addressed prevalence of poor sleep quality in patients on hemodialysis and its correlates. Reported prevalence of poor sleep quality has been found to be 34–78% in hemodialysis patients in majority of the studies [6–14]. Studies in pre-dialysis CKD patients are even limited with reported prevalence of 14–57% [1,13,15–17]. Fewer studies have compared quality of sleep between pre-dialysis CKD patients and ESRD [13,16,18–20] and have found inconsistent results with some studies showing no difference [13,16], while others showing higher frequency of poor sleep quality or sleep related problems in ESRD patients [18–20].

In a developing country like Pakistan, patients are often diagnosed with CKD at a later stage [21]. Due to late diagnosis of patients with CKD, there may not be any difference between quality of sleep between pre-dialysis CKD patients and ESRD patients. The objective of this study is to compare the prevalence of poor sleep quality between patients with CKD not on hemodialysis and ESRD in a developing country.

## Methods

This study was conducted in an out-patient department and hemodialysis unit of a tertiary care facility. Study was approved by institutional review board. Informed consent was obtained from all patients. Patients included in the study were above 18 years of age. Sample size was estimated to be 150 based on estimated prevalence of poor sleep quality as 50% with 95% confidence and 8% precision. Patients included had either chronic kidney disease, not on hemodialysis (pre-dialysis CKD) or patients who had end-stage renal disease on hemodialysis (ESRD). Pre-dialysis CKD was defined as estimated glomerular filtration rate (eGFR) of less than <60 mL/min/1.73m^2^ but excluding patients on renal replacement therapy [22]. eGFR was calculated by CKD-EPI formula [23].

We obtained patients’ history and laboratory data. We collected information on following variables: age, sex, height, weight, address, educational level, socio-economic, marital, employment, smoking status, duration of renal disease, hypertension, diabetes mellitus, cardiovascular disease, hepatitis C, blood hemoglobin, serum calcium, phosphorous, albumin and serum creatinine.

Cardiovascular disease was defined as known prior history of coronary artery disease, cerebrovascular disease or peripheral vascular disease based on history and review of prior medical records. Patient was considered to be in lower income class if daily income was less than twice the upper limit of accepted poverty line, i.e., $2 per household person [24]. Hypertension and/or Diabetes mellitus were defined as patients receiving such diagnoses by a health care provider and/or receiving treatment for either disease. A smoker was defined according to categories from the US Centers for Disease Control and Prevention, with current smokers being those adults who have smoked 100 cigarettes in their lifetime and currently smoke cigarettes every day (daily) or some days (nondaily) [25]. Weight was measured using a manual scale with accuracy up to 0.5 kg. Height was measured on barefoot patients using a fixed stadiometer with the measurement taken to the nearest 0.1 cm. Body mass index (BMI) was calculated as weight in kilograms divided by height in meters square. Serum creatinine was measured by Jaffe method using a kit by AMP Diagnostics, Austria. Hepatitis C was tested using third-generation enzyme-linked immunosorbent assay (ELISA).

Assessment of quality of sleep was done using Pittsburgh sleep quality index (PSQI). The Pittsburgh Sleep Quality Index (PSQI) is a self-reported questionnaire that consists of 19 individual items, creating 7 components that produce once global score ranging from 0 to 21. A lower score indicates healthier sleep quality. A score of 5 or greater is indicative of “poor sleep quality”. PSQI has been found to be valid and reliable in the assessment of self-reported sleep problems compared with actigraphic measures [26].

The level of anxiety and depression was assessed using hospital anxiety and depression scale (HADS) [27]. This scale is a self-reported questionnaire that consists of 14 individual items with half of these items evaluates anxiety, while remaining half evaluates depression. Total score for each anxiety and depression is between 0 and 21. A score of 8–10 is considered “borderline abnormal” and 11–21 is considered as “abnormal”.

### Statistical analysis

All statistical analyses were performed using SPSS 20.0 (SPSS Inc., Chicago, IL). Continuous parametric variables were reported as means ± standard deviation; non-parametric continuous variables were reported as median with 25–75 inter quartile range; and categorical variables were expressed as percentages. Normality was assessed by using QQ plots. Categorical variables were compared using the chi-square test, and continuous variables were compared using *t*-test for parametric data and Mann–Whitney *U*-test for non-parametric data. Spearman correlation was used to evaluate association between PSQI global score and eGFR in patients with pre-dialysis CKD. Since there were significant differences in several variables between pre-dialysis CKD and ESRD groups, we conducted a multiple linear regression analysis to study unadjusted and adjusted association between various predictor variables and PSQI global score in all patients. We included pre-dialysis CKD versus ESRD as a predictor variable in multiple linear regression analysis. Final model included demographic variables, pre-dialysis CKD versus ESRD and variables with an unadjusted *p* value of .10 or less. For all tests, *p* values of <.05 were considered statistically significant.

## Results

A total of 152 patients were included in the study. Mean age of all patients was 47.3 ± 18.3 years, 93 (61.2%) were males, and 59 (38.8%) were females. Out of these patients, 79 (52%) had ESRD and 73 (48%) had pre-dialysis CKD. Median duration of renal disease was 16 months (IQR 8–36 months). In patients with pre-dialysis CKD, median eGFR was 17.6 mL/min/1.73 m^2^ (IQR 7.8–24.5). Of these patients, 15.8% had stage III, 43.9% had stage IV, and 40.4% had stage V CKD. There was no significant co-relation between eGFR and global PSQI score (*r* = −0.34, *p* values .798) in pre-dialysis CKD patients.

Hypertension, diabetes mellitus, cardiovascular disease and hepatitis C were present in 81.6%, 46.7%, 19.7% and 28.3% of all patients, respectively. Mean hemoglobin was 9.8 ± 1.6 g/dl and median serum albumin was 3.9 g/dl (IQR 3.4-4.3 g/dl). Median PSQI score was 6 (IQR 3–8.8). Poor sleep quality (PSQI ≥5) was present in 100 (65.8%) patients.

A comparison of characteristics of patients with pre-dialysis CKD and ESRD is shown in Table 1. Patients with pre-dialysis CKD were older, more likely to be married, diabetic and resident of urban area, had better socioeconomic status, higher hemoglobin, lower phosphorous and lower frequency of hepatitis C.

**Table 1.** A comparison of characteristics of patients with pre-dialysis CKD and ESRD.

	Chronic kidney disease not on hemodialysis (pre-dialysis CKD), *N* = 73	End-stage renal disease on hemodialysis (ESRD), *N* = 79	*p* Value
Mean age in years	54.6 ± 16.7	40.6 ± 17.2	<.001
Male sex (%)	54.8	67.1	.120
Urban Address (%)	57.5	42.1	.060
Secondary or higher education (%)	47.2	52.8	.855
Middle or high socioeconomic status (%)	48.6	33.3	.057
Married (%)	88.9	70.9	.006
Working (%)	26.4	33.3	.354
Smoker (%)	11.1	6.3	.295
Median duration of renal disease in months (IQR)	12 (6–24)	18 (9–36)	.201
Hypertension (%)	83.6	79.7	.544
Diabetes mellitus (%)	60.3	36.5	.004
Cardiovascular disease (%)	23.6	17.1	.325
Hepatitis C (%)	11	44.9	<.001
Mean hemoglobin (g/dl)	10.3 ± 2.3	9.5 ± 1.6	.030
Median calcium (mg/dl) (IQR)	8.6 (8.1–9.0)	9.0 (8.6–9.6)	.987
Mean phosphorous (mg/dl)	5.5 ± 1.5	6.4 ± 1.6	.007
Median albumin (g/dl)	4.1 (3.5–4.3)	3.4 (1.7–3.9)	.009
Median body mass index (kg/m^2^)	21.7 (17.3–30.8)	19.1 (15.6–23.6)	.010

Table 2 depicts comparison of PSQI global scores and poor sleepers between pre-dialysis CKD and ESRD patients. There was no difference between the two groups. Multiple linear regression analysis of association of predictor variables with PSQI global scores for whole study population is shown in Table 3. We included pre-dialysis CKD versus ESRD as a predictor variable along with potential confounding variables which can affect PSQI. In the final model, only hemoglobin, depression and history of cardiovascular disease were associated with PSQI global score. Of note, there was no significant association between ESRD versus pre-dialysis CKD and PSQI global score.

**Table 2.** Comparison of PSQI global score between pre-dialysis CKD and ESRD patients.

	Chronic kidney disease not on hemodialysis (pre-dialysis CKD), *N* = 73	End-stage renal disease on hemodialysis (ESRD), *N* = 79	*p* Value
Median PSQI Global Score (IQR)	6 (3.5–9.0)	5 (3.0–8.0)	.381
Patients with poor sleep quality (%)	69.9%	62.0	.309

**Table 3.** Unadjusted and adjusted multiple linear regression analysis of predictors including ESRD versus pre-dialysis CKD of PSQI global score.

	Unadjusted Standardized coefficient *β*	*p* Value	Adjusted standardized coefficient *β*	Adjusted *p* value
Age	−0.052	.527		
Male sex	−0.078	.342		
Urban address (%)	−0.166	.043		
Secondary or higher education (%)	−0.110	.182		
Middle or high socioeconomic status (%)	−0.134	.102		
Married (%)	0.028	.730		
Working (%)	−0.158	.053		
Smoker (%)	0.115	.158		
Median duration of renal disease	−0.053	.557		
Hypertension (%)	0.173	.033		
Diabetes mellitus (%)	0.065	.434		
Cardiovascular disease (%)	0.207	.012	0.200	.025
Hepatitis C	0.008	.920		
Mean hemoglobin (g/dl)	−0.199	.027	−0.375	<.001
Median calcium (mg/dl)	0.122	.247		
Mean phosphorous (mg/dl)	−0.177	.078		
Median albumin (g/dl)	−0.093	.382		
Median body mass index (Kg/m2)	0.002	.979		
ESRD versus pre-dialysis CKD	−0.058	.480		
Anxiety score	0.276	.001		
Depression score	0.461	.001	0.505	<.001

Age, Sex, Address, socio-economic status, employment status, hypertension, cardiovascular disease, hemoglobin, phosphorous, anxiety, depression scores and ESRD vs. pre-dialysis CKD were included in final model in stepwise fashion.

## Discussion

In our study, we found that poor sleep quality was present in two-third of patients with CKD including patients on hemodialysis. There was no significant difference between quality of sleep between non-dialysis CKD patients and patients on hemodialysis after adjustment of other variables.

Our study results are comparable with other studies. Frequency of poor sleep quality was found in 57–78.5% in various studies involving hemodialysis patients [6–12]. Similarly, among CKD patients, poor sleep quality was found in 55.2–57% [16,17]. Few studies have shown significantly higher prevalence (84.7–87%) of poor sleepers among CKD and hemodialysis patients [28,29]. Other studies have shown much lower frequency. Poor sleep quality was found in 43.5% of patient on peritoneal dialysis [30]. In other studies, prevalence was found to be 34–49% among hemodialysis patients [13,14] and 14–47% among patients with chronic kidney disease [1,13,15]. Difference in study results can be explained by difference in patient population, geographic locations and methods of assessment of quality of sleep.

In this study, we found no difference in sleep quality between patients with chronic kidney disease not on hemodialysis and hemodialysis patients even after adjusting for other variables. In addition, we found no significant co-relation between eGFR and quality of sleep in patients with CKD not on hemodialysis. This finding can be explained by the fact that our patients had advanced CKD with 40.8% of patients had stage V CKD. This is consistent with late presentation of patients with CKD in our health care set up [21]. Few studies have assessed quality of sleep in both CKD and ESRD patients [13,16,18–20] and have shown variable results, which may in part can be explained by different assessment tools used in these studies to assess sleep quality. Agarwal et al. [18] found that patients on hemodialysis had more disrupted sleep but there was no association between eGFR and sleep disruption in CKD patients. In a study by Roumelioti et al. [19], dialysis dependency was associated with poor sleep quality. Kurella et al. and Kumar et al. found no difference in sleep quality between CKD and ESRD patients like in our study [13,16], although significant correlation between eGFR and quality of sleep was found in the study by Kurella et al. [13]. In another study, hemodialysis patients with sleep disordered breathing were found to have poor sleep quality compared with those not on hemodialysis [20]. However, this study only assessed sleep quality in patients who had sleep-disordered breathing.

In our study, we found that history of cardiovascular disease, high-depression score and lower hemoglobin were associated with poor sleep quality in a multivariate analysis. Association between lower hemoglobin and poor sleep quality was found in other studies [8,31]. Depression was found to be associated with poor sleep quality in several studies [16,30–34]. History of heart failure was also found to be associated with poor sleep quality in another study [34]. Multiple other predictors of poor sleep quality have been found in other studies but not in our study including young age [7,12,16,19], older age [11,13,34], female sex [11,15,31,35], white race [19], employment [7], diabetes mellitus [6], duration of hemodialysis [12,30], reduced frequency of hemodialysis [11], body pain [6,16], pruritis [16], CRP [7], serum phosphorous [7,8,15,32], albumin, body mass index [7] and quality of life [9,12,19,20,29]. Difference in predictors of poor sleep quality is likely explained by difference in patient population, sample size, variation in inclusion of predictor variables and assessment tools for sleep quality.

Poor sleep quality may have several implications. Poor sleep quality has been found to be associated with increased mortality [14] in hemodialysis patients and higher risk of mortality in pre-dialysis CKD patients [16], hospital stay [12], non-dipping hypertensive pattern [36] and periventricular white matter intensity in elderly CKD patients [37].

Our study has several limitations. This is a single center study with sizable but still limited sample size. We have assessed substantial but not all possible variables which could be associated with poor sleep quality. In addition, we do not have a longitudinal follow up of patients to examine impact of poor sleep quality on patient outcomes.

In summary, poor sleep quality is common in patients with CKD including hemodialysis patients in a developing country, which is independent of kidney function in pre-dialysis patients. There is no difference in quality of sleep between patients on hemodialysis and pre-dialysis CKD patients even after adjustment of confounding variables. Clinicians taking care of these patients should routinely assess and address sleep quality in these patients including those not yet on dialysis. Further studies are needed to evaluate impact of sleep quality on risk of hospitalization, end-stage renal disease and mortality in our patient population.
